# Factors related to medication errors in neonatal intensive care in Saudi Arabia: A nationwide survey

**DOI:** 10.1097/MD.0000000000047470

**Published:** 2026-01-30

**Authors:** Hassan Al-shehri, Adel Abed AlHarbi, Ghadah Saad Algoraini, Ghayda Khaled Aldosari, Ghaida Majed Almasari, Fatimah Abdullah Aljawad, Kadi Saud Alsubaie, Zainab Moneer Altarouti

**Affiliations:** a Department of Pediatrics, College of Medicine, Imam Mohammad Ibn Saud Islamic University (IMSIU), Riyadh, Saudi Arabia; b Division of Neonatology, Department of Pediatrics, Prince Sultan Military Medical City, Riyadh, Saudi Arabia; c College of Medicine, Imam Mohammad Ibn Saud Islamic University (IMSIU), Riyadh, Saudi Arabia.

**Keywords:** medication errors, neonatal care, neonatal intensive care units (NICUs), prescribing errors, Saudi Arabia

## Abstract

Neonates are at higher risk of experiencing medication errors. Limited research has thoroughly examined the underlying factors related to these errors from nurses’ perspective, who are essential in medication management within Neonatal intensive care unit (NICU)s. The aim of this study was to characterize medication errors in NICU across Saudi Arabia and to categorize the types of errors. This is an online cross-sectional survey study that was conducted in Saudi Arabia between February 2024 and August 2024. The study population consisted of nurses working in the NICU. The questionnaire was designed to assess medication errors in NICU settings. A total of 142 NICU nurses participated in this study. Prescribing errors were the most common, accounting for 36.6% of reported cases, followed by medication preparation errors (24.6%) and dispensing errors (15.5%). Nurses with 1 to 5 years of experience had the highest error rates, particularly in prescribing and preparation. Level 4 NICUs reported higher error rates during these stages. Higher nurse-to-infant ratios was associated with higher medication error rates (*P* = .042), highlighting the impact of workload on safety. This study underscores the need for targeted interventions, such as improved dosing protocols, training for less experienced nurses, and strategies to optimize nurse-to-infant ratios in NICUs. Future research should evaluate the effectiveness of these interventions in reducing medication errors to enhance neonatal care outcomes in Saudi Arabia and similar settings.

## 1. Introduction

Medication errors are defined as “any avoidable incident that may lead to drug overuse or patient harm,” which are considered as significant and preventable category of medical errors.^[1,2]^ Patients with life-threatening clinical conditions who undergo various pharmacological treatments and interventions face an elevated chance of medication errors.^[3]^

The first days of life for neonates are considered a highly vulnerable period for them, rendering it the most critical phase of life.^[4,5]^ Neonates receive specialized care once they admitted to the neonatal intensive care unit (NICU).^[6]^ After admitting neonates to NICU, they become vulnerable to medication errors due to the various types of intervention that they become exposed to including the administration of multiple medications and performing other procedures essential for their critical conditions and lives.^[7]^ Based on the level of care provided, NICUs are categorized into 4 levels^[8]^; which are Level I which offers general care for healthy late preterm and full-term infants, Level II which offers care for moderately ill or premature infants (≥32 weeks) who need close monitoring, Level III which has advanced care for very preterm or severely ill infants, and Level IV which is the most advanced and delivers specialized care to the most complex cases.^[8]^

Neonates are at higher risk of experiencing medication errors and this risk might reach eightfold in comparison to what observed in adult hospital settings.^[9]^ Use of prescription medications within the NICU undergo dramatic changes in term of dose and interval range and is influenced by multiple factors including birth weight, gestational age, and postnatal age.^[7]^ Besides, the rate of medication errors in the NICU is estimated to be around 13 to 91 per 100 admissions.^[10,11]^ Most of neonates’ death incidents occur during the early neonatal period, which is a crucial stage for the prevention, management, and observation of complications associated with delivery.^[12]^ Through the implementation of recommended neonatal care practices around 70% of neonatal deaths could be prevented, as these practical measures proofed its effectiveness in declining newborn mortality rates.^[12]^

Recent research showed rising trends of hospitalization due to medication errors for patients from all age groups.^[13–16]^ Limited research has thoroughly examined the underlying factors related to these errors from nurses’ perspective, who are essential in medication management within NICUs in Saudi Arabia. Besides, due to the increasing complexity of neonatal pharmacotherapy the aim of this study was to characterize medication errors in NICUs across Saudi Arabia and to categorize the types of errors.

## 2. Materials and methods

### 2.1. Study design

This is an online cross-sectional survey study that was conducted in Saudi Arabia between February 2024 and August 2024.

### 2.2. Sample selection

The convenience sampling technique was used to recruit the study sample, this sampling technique involves selecting easily accessible participants, thereby allowing for rapid data collection. Convenience sampling offers the advantage of simplicity, as it does not require complex sampling frames.

### 2.3. Study population

The study population consisted of nurses working in the NICU, regardless of the level of care provided in that specific NICU in Saudi Arabia. There was no restriction on years of experience, NICU practice settings, or infant to nurse ratio in the practice settings for the study participants. Nurses with no previous experience in the NICU were excluded.

### 2.4. Data collection

Nurses working in NICUs nationwide were invited to participate in the survey. Data were collected using a Google Form questionnaire distributed via WhatsApp, email, Twitter, and Telegram. Data collection was limited to a 1-time distribution and regular reminders through digital platforms (WhatsApp and emails), with no follow-up, to minimize respondent burden and ensure voluntary participation. Each nurse was provided detailed information about the study’s objectives, procedures, potential risks, and benefits. Participation was entirely voluntary, and participants were assured of their right to withdraw from the study at any time without any repercussions. Confidentiality and anonymity of the participants were strictly maintained throughout the study, with all data being securely stored and accessible only to the research team.

### 2.5. Survey instrument

The questionnaire was designed to assess medication errors in NICU settings. Additionally, information was gathered regarding the most commonly used medications in NICU practice. The survey instrument comprised 13 questions, including multiple-choice and yes/no formats. The questionnaire was divided into 3 sections. The first section addressed the participants’ practice characteristics, such as their role as neonatal intensive care nurses, years of experience, and the specific NICU level in which they were employed. The second section focused on the operational aspects of medication management within the NICU, including the nurse-to-infant ratio, the use of standardized protocols for medication management, the implementation of barcode scanning and electronic medication administration records (eMAR), the adoption of double-checking systems for high-risk medications, and staff education on medication safety. The third section contained questions related to the characteristics of medication errors and the stages of the medication process most prone to errors.

### 2.6. Questionnaire piloting

Expert neonatologists reviewed the proposed questionnaire to ensure compliance with the external validity standards. The researchers evaluated the validity of the content to ensure that the questionnaire items covered the constructed area being studied. Another group of expert neonatologists and NICU nurses performed a subsequent evaluation. Following these evaluations, the questionnaire was tested in a pilot study involving a group of NICU nurses. The findings of the pilot study confirmed that the questions accurately reflected the participants’ clinical practice and were easily understandable.

### 2.7. Ethical approval

Informed consent was obtained from all participants before their inclusion in the study. The Al-Imam Mohammad Ibn Saud Islamic University institutional review board approved this study in Riyadh, Saudi Arabia (Project number 605/2024). All patients provided their written consent before participating in the study.

### 2.8. Sample size

This is an exploratory study; therefore, no formal a priori sample size calculation was performed.

### 2.9. Statistical analysis

Data were analyzed using the Statistical Package for Social Science Software (SPSS, Chicago) version 29. Demographic variables and NICU-related categorical variables were summarized using frequency and percentage distributions. A chi-square test/ Fisher Exact Test was conducted to examine the statistically significant difference in proportion of different types of medication errors in terms of nurse/NICU-related factors. The significance level was assigned as *P*-value <.05.

## 3. Results

### 3.1. NICU settings and nurse attributes

A total of 142 NICU nurses participated in this study. The distribution of NICU levels shows that Level 4 NICUs are the most common (29.6%), followed by Level 3 (28.2%), Level 1 (26.8%), and Level 2 (15.5%). All respondents were neonatal intensive care nurses. The nurse-to-infant ratio varies, with the majority (53.5%) having a ratio of 1 nurse to 2 infants, followed by 1 nurse to 3 infants (23.2%), more than 3 infants per nurse (12.7%), and 1 nurse to 1 infant (10.6%). Nurses’ work experience ranges widely, with the largest group (41.5%) having 1 to 5 years of experience, followed by those with 6 to 10 years (32.4%), and smaller groups with 11 to 20 years (20.4%) and more than 20 years (5.6%). The overview of the NICU settings and the attributes of neonatal intensive care nurses are provided in Table 1.

**Table 1:** Distribution of basic characteristics of nurses and NICU.

Variables	Options	Frequency	Percentage
NICU practicing settings	Level 1 NICU	38	26.8
	Level 2 NICU	22	15.5
	Level 3 NICU	40	28.2
	Level 4 NICU	42	29.6
Infant to nurse ratio	One nurse to 1 infant	15	10.6
	One Nurse to 2 infants	76	53.5
	One Nurse to 3 infants	33	23.2
	One Nurse to more than 3 infants	18	12.7
Year of experiences	1–5 yr	59	41.5
	11–20 ys	29	20.4
	6–10 yr	46	32.4
	More than 20 yr	8	5.6

NICU = neonatal intensive care unit.

### 3.2. NICU standardized protocols and safety practices

Standardized protocols and safety practices within various NICU settings were analyzed. The vast majority of NICUs nurses (97.2%) reported that their practicing sites have standardized protocols and guidelines. Over half (53.5%) use barcode scanning and electronic medication administration. Nearly all NICUs have clear drug reconciliation procedures (94.4%), and most (93%) have a system for double-checking medications. Additionally, 90.8% of NICUs provide staff education and training on medication safety, Table 2.

**Table 2:** Distribution of responses from nurses regarding standardized protocols in NICU.

	Frequencies	Percentage
Has your NICU had standardized protocols and guidelines?		
No	4	2.8
Yes	138	97.2
Is your NICU using barcode scanning and electronic medication administration?		
No	66	46.5
Yes	76	53.5
Is there a clear process for medication reconciliation?		
No	8	5.6
Yes	134	94.4
Is your unit implementing a system of double-checking?		
No	10	7.0
Yes	132	93.0
Do you have staff education and training on medication safety?		
No	13	9.2
Yes	129	90.8

NICU = neonatal intensive care unit.

### 3.3. Distribution of medication errors in NICU settings

The distribution of medication errors in NICU settings was analyzed. Prescribing errors are the most common, accounting for 36.6% of medication errors, followed by medication preparation errors (24.6%) and dispensing errors (15.5%). Other type of medication errors, such as administration and transcription errors, were less frequent. Monitoring errors after medication administration are the least common (3.5%). This analysis describes the different types of medication errors that occur within the NICU.

It was observed that the most common form of medication error was coming from prescribing errors (36.6%), followed by medication preparation errors (24.6%), and dispensing errors (15.5%), Table 3.

**Table 3:** Distribution of different kinds of Medication errors at NICU.

Most medication errors occur at which stage of the medication process	Frequencies	Percentage
Prescribing error	52	36.6
Medication preparation errors	35	24.6
Dispensing errors	22	15.5
Administration errors	14	9.9
Transcription errors	14	9.9
After administering a medication (monitoring errors)	5	3.5

NICU = neonatal intensive care unit.

### 3.4. Medication errors stratified by nurse and NICU settings

Table 4 below shows medication errors stratified by nurse experience and NICU Settings. Nurses with 1 to 5 years of experience had the highest incidence of errors, particularly in prescribing (42.3%) and medication preparation (40%). Those with 6 to 10 years of experience also recorded significant error rates, especially in dispensing (50%). Level 4 NICUs experienced higher error rates during the prescribing and drug preparation stages compared to other levels. The nurse-to-infant ratio showed statistically significant impacted on medication error rates, with higher ratios (e.g., 1 nurse to 2 infants) associated with higher prescribing (65.4%) and medication preparation errors rates (54.3%). Conversely, lower error rates were observed among nurses with more experience and a 1:1 nurse-to-infant ratio. This data underscores the importance of experience, NICU level, and workload in influencing medication safety, with a significant correlation in the nurse-to-infant ratio (*P* = .042).

**Table 4:** Medication errors stratified by nurse experience and NICU settings.

Variable	Administration errors	After administering a medication (monitoring errors)	Dispensing errors	Medication preparation errors	Prescribing error	Transcription errors	Total	*P*-value
Year of experiences								
1–5 yr	7 (50)	5 (100)	6 (27.3)	14 (40)	22 (42.3)	5 (37.5)	59 (41.5)	.186
11–20 yr	4 (28.6)	0 (0)	3 (13.6)	10 (28.6)	9 (17.3)	3 (21.4)	29 (20.4)	
6–10 yr	2 (14.3)	0 (0)	11 (50)	11 (31.4)	16 (30.8)	6 (42.9)	46 (32.4)	
More than 20 yr	1 (7.1)	0 (0)	2 (9.1)	0 (0)	5 (9.6)	0 (0)	8 (5.6)	
NICU practicing settings								
Level 1 NICU	3 (21.4)	2 (40)	6 (27.3)	9 (25.7)	11 (21.2)	7 (50)	38 (26.8)	.525
Level 2 NICU	1 (7.1 )	2 (40)	5 (22.7)	6 (17.1)	6 (11.5)	2 (14.3)	22 (15.5)	
Level 3 NICU	6 (42.9)	0 (0)	4 (18.2)	9 (25.7)	19 (36.5)	2 (14.3)	40 (28.2)	
Level 4 NICU	4 (28.6)	1 (20)	7 (31.8)	11 (31.4)	16 (30.8)	3 (21.4)	42 (29.6)	
Nurse-to-infant ratio								
One nurse to 1 infant	3 (21.4)	0 (0)	0 (0)	4 (11.4)	6 (11.5)	2 (14.3)	15 (10.6)	**.042**
One Nurse to 2 infants	6 (42.9)	2 (40)	8 (36.4)	19 (54.3)	34 (65.4)	7 (50)	76 (53.5)	
One Nurse to 3 infants	4 (28.6)	1 (20)	12 (54.5)	7 (20)	5 (9.6)	4 (28.6)	33 (23.2)	
One Nurse to more than 3 infants	1 (7.1)	2 (40)	2 (9.1)	5 (14.3)	7 (13.5)	1 (7.1)	18 (12.7)	

NICU = neonatal intensive care unit.

### 3.5. Common medications used in NICU practice

The most commonly used medications in NICU practice have been illustrated in Table 5. Antibiotics are the most frequently used, accounting for 57.7% of total medication usage. Intravenous fluids and electrolytes are the next most common (28.2%). Other medications, such as inotropes (3.5%) and sedatives/analgesics (2.8%), are less commonly used. In comparison, only 0.7% indicated that the choice of medication depends on the specific case or involves both antibiotics and fluids with electrolytes. This data indicates a firm reliance on antibiotics in NICU settings.

**Table 5:** Most common medicat+ion categories that are used in NICU practice.

Most common medication categories that are used in your NICU practice	Frequencies	Percentage
Antibiotics	82	57.7
Intravenous fluids and electrolytes	40	28.2
Inotropes	5	3.5
Sedatives and analgesics	4	2.8
Both antibiotics and fluids with electrolytes	1	0.7
Depends upon the case of the baby	1	0.7

NICU = neonatal intensive care unit.

## 4. Discussion

This study examined the characteristics of medication errors in NICUs in Saudi Arabia, focusing on the perspectives of nurses. Our findings indicate that prescribing errors are the most common medication errors, accounting for 36.6% of all reported cases. These findings were in line with other previous studies as Pawluk et al (2017) in Qatar identified that most type of medication errors (98.5%) in the NICU occurred during the prescribing phase of which 58.9% were related to calculation errors.^[17]^ Similarly, in Iran, Eslami et al (2019) identified that the most common 2 causes of medication errors were wrong dosage by physicians in prescription phase (28%) and not administering medication by nurse in administration phase (29%).^[7]^ A previous systematic review found medication error rates in NICUs ranged from 4 to 35.1 per 1000 patient-days and from 5.5 to 77.9 per 100 medication orders.^[18]^ Furthermore, prescribing and medication administration errors were identified as the most common medication errors, with dosing errors the most frequently reported error subtype.^[18]^ There are multiple factors that might contribute to the incidence of prescribing errors, these include workload, work environment, whether or not physicians were prescribing for their own patient, communication within their team, and lack of knowledge.^[19]^ Besides, other important influencing factors include organizational factors such as inadequate training, a hierarchical medical team, and an absence of self-awareness of errors.^[19]^

In this study, staffing levels significantly impacted the frequency and nature of medication errors, as nurse-to-infant ratio showed statistically significant impacted on medication error rates, with higher ratios (e.g., 1 nurse to 2 infants) associated with higher prescribing (65.4%) and medication preparation errors (MAE) rates (54.3%)(*P* = .042). A previous study by Henry Basil, J. et al found that the most common reasons for MAEs were inadequate nursing staff.^[20]^ Other identified causes of MAEs include having error-provoking conditions which have high influence on administration errors which include inadequate written communication (whether documentation, prescriptions, or transcription), having problems with medicines supply and storage, high perceived workload, and staff health status (fatigue, stress).^[21]^

In our study, we found that Level 4 NICUs experienced higher medication error rates, specifically prescribing errors and drug preparation errors. This aligns with previous research by Stratton et al which reported that in appropriate nurse to patient’s ratio was a main contributing factor for medication errors.^[22]^ Due to the nature of high workload within the NICUs, high nurse to infant ratio and inadequate staffing might allocated time for patient care, which ultimately might increase the rate of medication errors.

In our study, we found that antibiotics (57.7%) and intravenous fluids and electrolytes (28.2%) are the most frequently used medications in the NICUs. These 2 types of medications are commonly used due to their vital role in managing antimicrobial infections and maintaining fluid and electrolyte balance.

Furthermore, we found that the vast majority of NICUs nurses (97.2%) reported that their practicing sites have standardized protocols and guidelines. NICU nurses are required to have expertise in a wide range of complicated and life-threatening tasks in order to respond to the rapidly changing health conditions of neonates.^[23]^ Besides, NICU nurses are required to have timely and accurate clinical decision-making skills for neonates with different health conditions.^[23]^ NICU nurses’ clinical decision-making skills and practices increases the quality of nursing and have positive influence on neonates treatment outcomes.^[24]^ Therefore, it is crucial for the nurses to have standard guidelines for practice.^[25]^ In clinical situations, standard guidelines for practice help to improve nurses’ competency.^[26]^

In our study, we found that over half (53.5%) use barcode scanning and electronic medication administration. This becomes more commonly integrated with electronic prescribing and medication administration (ePMA) systems in many countries.^[27]^ Barcode medication administration has multiple patient safety benefits, which include reducing both the rate and severity of MAEs.^[28–30]^

In our study, nearly all NICUs have clear drug reconciliation procedures (94.4%), and most (93%) have a system for double-checking medications. Medication reconciliation is defined as “the process of comparing a patient’s medication orders to all of the medications that the patient has been taking.”^[31]^ The main purpose of implementing medication reconciliation is to avoid medication errors. Therefore, medication reconciliation should be applied at each and every transition of healthcare stage in which new medications are ordered or existing orders are rewritten.^[31]^ Transitions in healthcare include changes in practitioner, level of care, setting, or service.^[31]^

This study has limitations. This is an online survey study, which might have affected the generalizability of our study findings. The use of cross-sectional study design restricted the ability to examine causality among the study variables. This research used self-administered questionnaire which might increase the possibility of reporting bias. Besides, the study’s reliance on nurse perceptions rather than objective data, which undermines its ability to identify true causative factors. Furthermore, self-reported data is prone to social desirability bias. Therefore, our findings should be interpreted carefully. Future studies should examine MAEs among different settings and apply follow-up in order to examine causality among study variables. Furthermore, more studies are needed to examine MAEs from different perspectives including physician, pharmacists within NICUs. Furthermore, future studies could benefit from triangulating nurse perceptions with objective clinical data.

## 5. Conclusion

The findings of this study revealed that prescribing errors are the most prevalent type of medication error within NICUs, followed by medication preparation, and dispensing errors. Furthermore, this study identified that nurse staffing levels significantly impact medication error rates with higher nurse-to-infant ratios associated with higher MAEs rates. Therefore, we recommend implementing more strict practices within NICUs in order to enhance patients’ safety and decrease the probability of MAEs. Additionally, optimizing nurse-to-infant ratios and ensuring adequate support for less experienced nurses can help mitigate the risk of medication errors.

## Acknowledgments

This work was supported and funded by the Deanship of Scientific Research at Imam Mohammad Ibn Saud Islamic University (IMSIU) (grant number IMSIU-DDRSP2601).

## Author contributions

**Conceptualization:** Hassan Al-shehri.

**Data curation:** Hassan Al-shehri.

**Formal analysis:** Hassan Al-shehri.

**Funding acquisition:** Hassan Al-shehri.

**Investigation:** Hassan Al-shehri, Adel Abed AlHarbi, Ghadah Saad Algoraini, Ghayda Khaled Aldosari, Ghaida Majed Almasari, Fatimah Abdullah Aljawad, Kadi Saud Alsubaie, Zainab Moneer Altarouti.

**Methodology:** Hassan Al-shehri.

**Project administration:** Hassan Al-shehri.

**Resources:** Hassan Al-shehri, Adel Abed AlHarbi, Ghadah Saad Algoraini, Ghayda Khaled Aldosari, Ghaida Majed Almasari, Fatimah Abdullah Aljawad, Kadi Saud Alsubaie, Zainab Moneer Altarouti.

**Software:** Hassan Al-shehri.

**Supervision:** Hassan Al-shehri.

**Validation:** Hassan Al-shehri.

**Visualization:** Hassan Al-shehri.

**Writing – original draft:** Hassan Al-shehri.

**Writing – review & editing:** Hassan Al-shehri, Adel Abed AlHarbi, Ghadah Saad Algoraini, Ghayda Khaled Aldosari, Ghaida Majed Almasari, Fatimah Abdullah Aljawad, Kadi Saud Alsubaie, Zainab Moneer Altarouti.
